# *catena*-Poly[[bis­[4-(1*H*-imidazo[4,5-*f*][1,10]phenanthrolin-2-yl)phenol]cadmium(II)]-μ-fumarato]

**DOI:** 10.1107/S1600536809024246

**Published:** 2009-07-08

**Authors:** Li-Ping Shi, Edward R. T. Tiekink

**Affiliations:** a Department of Chemistry, College of Chemistry and Biology, Beihua University, Jilin City 132013, People’s Republic of China; b Universidade Federal de São Carlos, Laboratório de Cristalografia, Estereodinâmica e Modelagem Molecular, Departamento de Química, 13565-905 São Carlos, SP, Brazil

## Abstract

In the polymeric title compound, [Cd(C_4_H_2_O_4_)(C_19_H_12_N_4_O)_2_]_*n*_, the Cd^II^ centre is eight-coordinated within an N_4_O_4_ donor set derived from two chelating 4-(1*H*-imidazo[4,5-*f*][1,10]phenanthrolin-2-yl)phenol ligands and two asymmetrically chelating carboxyl­ate residues of bridging fumarate dianions. The linear chains are linked into a layer in the *ac* plane *via* O—H⋯O_carboxyl­ate_ hydrogen bonds. Layers are connected into double layers *via* N—H⋯O_carboxyl­ate_ hydrogen bonds and these stack along the *b* axis. C—H⋯π inter­actions are also present. Disorder in the ethyl­ene portion of the fumarate was modelled over two positions, the major component having a site-occupancy factor of 0.677 (15).

## Related literature

For general background and related structures see: Chen & Liu (2002 ▶); Yang *et al.* (2007*a* ▶,*b* ▶).
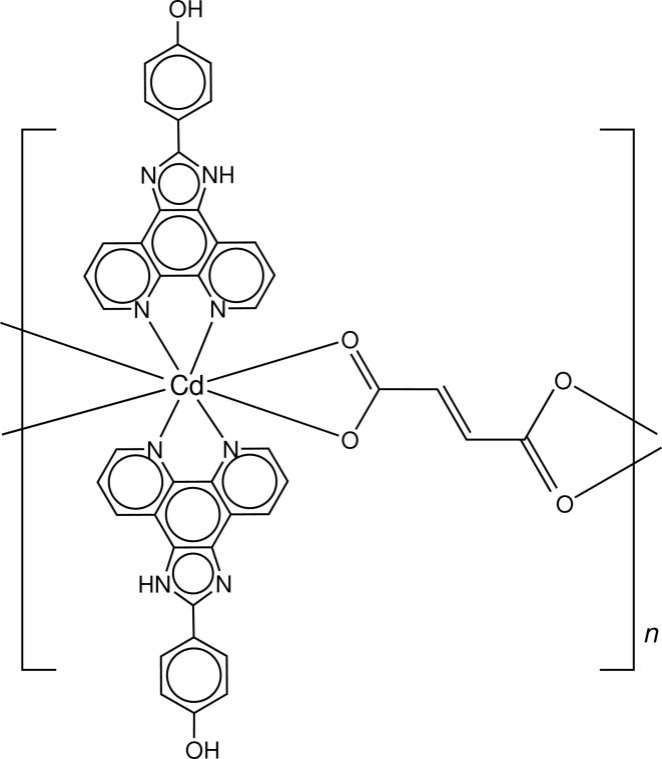


## Experimental

### 

#### Crystal data

[Cd(C_4_H_2_O_4_)(C_19_H_12_N_4_O)_2_]
*M* *_r_* = 851.11
Triclinic, 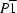

*a* = 9.5596 (3) Å
*b* = 13.5628 (7) Å
*c* = 15.8934 (16) Å
α = 64.756 (3)°
β = 77.142 (1)°
γ = 72.929 (4)°
*V* = 1770.4 (2) Å^3^
*Z* = 2
Mo *K*α radiation
μ = 0.68 mm^−1^
*T* = 293 K
0.33 × 0.25 × 0.20 mm

#### Data collection

Bruker SMART APEX diffractometer
Absorption correction: multi-scan (*SADABS*; Sheldrick, 1996 ▶) *T*_min_ = 0.654, *T*_max_ = 0.772 (expected range = 0.739–0.873)
15185 measured reflections
7170 independent reflections
5600 reflections with *I* > 2σ(*I*)
*R*_int_ = 0.028

#### Refinement

*R*[*F* ^2^ > 2σ(*F* ^2^)] = 0.043
*wR*(*F* ^2^) = 0.119
*S* = 1.05
7170 reflections
529 parameters
2 restraints
H-atom parameters constrained
Δρ_max_ = 1.67 e Å^−3^
Δρ_min_ = −0.30 e Å^−3^

### 

Data collection: *SMART* (Bruker, 1997 ▶); cell refinement: *SAINT* (Bruker, 1999 ▶); data reduction: *SAINT*; program(s) used to solve structure: *SHELXS97* (Sheldrick, 2008 ▶); program(s) used to refine structure: *SHELXL97* (Sheldrick, 2008 ▶); molecular graphics: *DIAMOND* (Brandenburg, 2006 ▶); software used to prepare material for publication: *SHELXL97*.

## Supplementary Material

Crystal structure: contains datablocks global, I. DOI: 10.1107/S1600536809024246/lh2849sup1.cif

Structure factors: contains datablocks I. DOI: 10.1107/S1600536809024246/lh2849Isup2.hkl

Additional supplementary materials:  crystallographic information; 3D view; checkCIF report

## Figures and Tables

**Table 1:** Hydrogen-bond geometry (Å, °)

*D*—H⋯*A*	*D*—H	H⋯*A*	*D*⋯*A*	*D*—H⋯*A*
O1—H1*O*⋯O6^i^	0.84	1.86	2.659 (5)	161
O2—H2*O*⋯O4^ii^	0.84	1.83	2.663 (5)	172
N3—H3*n*⋯O5^iii^	0.86	2.15	2.893 (6)	144
N7—H7*n*⋯O3^iv^	0.86	2.06	2.785 (6)	141
C3—H3⋯O5^iii^	0.93	2.48	3.309 (5)	148
C22—H22⋯O3^iv^	0.93	2.57	3.360 (5)	143
C28—H28⋯O2^v^	0.93	2.55	3.390 (6)	150
C2—H2⋯*Cg*1^iv^	0.93	2.75	3.445 (5)	133
C21—H21⋯*Cg*2^vi^	0.93	2.79	3.554 (5)	140

Symmetry codes: (i) 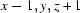; (ii) 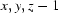; (iii) 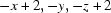; (iv) 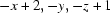; (v) 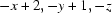; (vi) 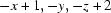. *Cg*1 and *Cg*2 are the centroids of the C33–C38 and C14–C19 rings, respectively.

**Fig. 1.**
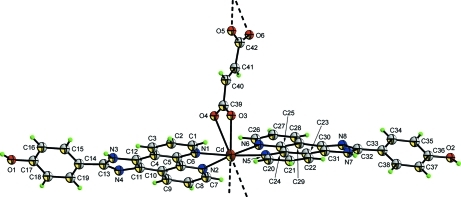
The asymmetric unit in the polymeric structure of (I), showing the atomic numbering scheme. Displacement ellipsoids are drawn at the 70% probability level. Only the major component of the disordered ethylene residue in the fumarate dianion is shown.

**Fig. 2.**
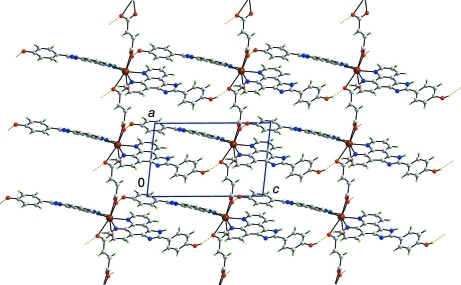
View of links mediated by O—H···O hydrogen bonding (dashed lines) between the polymeric chains in (I).

**Fig. 3.**
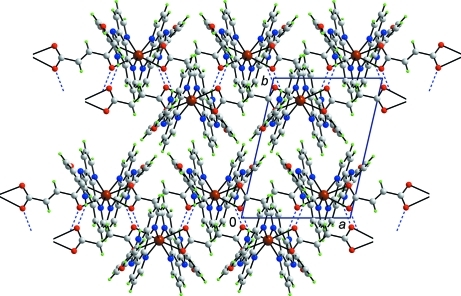
View of the double layers mediated by N—H···O hydrogen bonding (dashed lines) and the stacking of these in the crystal structure of (I).

## supplementary crystallographic information

### Comment

The chelating molecules 1,10-phenanthroline and 2,2'-bipyridyl have been widely used to build supramolecular architectures owing to their excellent coordinating ability and large conjugated system (Chen & Liu, 2002). However, far less attention has been given to their derivatives (Yang *et al.*, 2007*a*; Yang *et al.*, 2007*b*). For example, the rare phenanthroline derivative 4-(1*H*-imidazo[4,5-*f*][1,10]phenanthrolin-2-yl)phenol (*L*) possesses varied aromatic systems, and is a good candidate for the construction of metal-organic supramolecular architectures. In this contribution, a cadmium coordination polymer containing *L* and fumarate has been synthesized, namely [Cd*L*_2_(C_4_H_2_O_4_)]_n_ (I), and its crystal structure determined.

The asymmetric unit of (I) comprises cadmium, two chelating *L* ligands and a bridging fumarate dianion, Fig. 1. The Cd–N bond distances lie in the narrow range 2.320 (3) to 2.351 (3) Å and, reflecting the asymmetric mode of coordination exhibited by the carboxylate residues, the Cd–O distances range from 2.496 (3) to 2.742 (3) Å. The cadmium centre is eight-coordinate within an N_4_O_4_ donor set. The polymeric chain is linear, Fig. 2, and these form a layer in the *ac* plane with adjacent chains being connected by O–H···O hydrogen bonds, Table 1. Centrosymmetrically related layers associate *via* N—H···O hydrogen bonds to form a double layer and these aggregates stack along the *b* axis, Fig. 3. Further consolidation to the crystal packing is afforded by C–H···O interactions that occur within layers and between double layers, and by C–H···π contacts within double layers, Table 1.

### Experimental

A mixture of fumeric acid (0.5 mmol), [4-(1*H*-imidazo[4,5-*f*][1,10]phenanthrolin-2-yl)phenol] (0.5 mmol), NaOH (1 mmol) and CdCl_2_^.^2H_2_O (0.5 mmol) was suspended in deionized water (12 ml) and sealed in a 20-ml Teflon-lined autoclave. Upon heating at 433 K for one week, the autoclave was slowly cooled to room temperature. The crystals were collected, washed with deionized water, and dried.

### Refinement

Carbon-bound H-atoms were placed in calculated positions with C—H = 0.93 Å, and were included in the refinement in the riding model approximation, with *U*(H) set to 1.2*U*_eq_(C). The O—H and N—H atoms were located from a difference map but included in their idealized positions with O—H = 0.84 Å and *U*(H) set to 1.5*U*_eq_(O) and N—H = 0.86 Å and *U*(H) set to 1.2*U*_eq_(N).

Disorder was noted in the positions of the ethylene atoms of the fumarate dianion. The atoms were modelled over two positions with the major component (anisotropic displacement parameters) having a site occupancy = 0.677 (15).

The maximum and minimum residual electron density peaks of 1.67 and 0.30 e Å^-3^, respectively, were located 1.37 Å and 1.53 Å from the H21 and N2 atoms, respectively.

### Crystal data

**Table:**

[Cd(C_4_H_2_O_4_)(C_19_H_12_N_4_O)_2_]	*Z* = 2
*M**_r_* = 851.11	*F*(000) = 860
Triclinic, *P*1	*D*_x_ = 1.597 Mg m^−^^3^
Hall symbol: -P 1	Mo *K*α radiation, λ = 0.71073 Å
*a* = 9.5596 (3) Å	Cell parameters from 7163 reflections
*b* = 13.5628 (7) Å	θ = 3.0–26.4°
*c* = 15.8934 (16) Å	µ = 0.68 mm^−^^1^
α = 64.756 (3)°	*T* = 293 K
β = 77.142 (1)°	Block, pale-yellow
γ = 72.929 (4)°	0.33 × 0.25 × 0.20 mm
*V* = 1770.4 (2) Å^3^	

### Data collection

**Table:**

Bruker SMART APEX diffractometer	7170 independent reflections
Radiation source: fine-focus sealed tube	5600 reflections with *I* > 2σ(*I*)
graphite	*R*_int_ = 0.028
φ and ω scans	θ_max_ = 26.4°, θ_min_ = 4.3°
Absorption correction: multi-scan (*SADABS*; Sheldrick, 1996)	*h* = −8→11
*T*_min_ = 0.654, *T*_max_ = 0.772	*k* = −16→16
15185 measured reflections	*l* = −19→19

### Refinement

**Table:**

Refinement on *F*^2^	Primary atom site location: structure-invariant direct methods
Least-squares matrix: full	Secondary atom site location: difference Fourier map
*R*[*F*^2^ > 2σ(*F*^2^)] = 0.043	Hydrogen site location: inferred from neighbouring sites
*wR*(*F*^2^) = 0.119	H-atom parameters constrained
*S* = 1.05	*w* = 1/[σ^2^(*F*_o_^2^) + (0.0737*P*)^2^ + 0.2724*P*] where *P* = (*F*_o_^2^ + 2*F*_c_^2^)/3
7170 reflections	(Δ/σ)_max_ = 0.002
529 parameters	Δρ_max_ = 1.67 e Å^−^^3^
2 restraints	Δρ_min_ = −0.30 e Å^−^^3^

### Special details

**Table:**

Geometry. All s.u.'s (except the s.u. in the dihedral angle between two l.s. planes) are estimated using the full covariance matrix. The cell s.u.'s are taken into account individually in the estimation of s.u.'s in distances, angles and torsion angles; correlations between s.u.'s in cell parameters are only used when they are defined by crystal symmetry. An approximate (isotropic) treatment of cell s.u.'s is used for estimating s.u.'s involving l.s. planes.
Refinement. Refinement of *F*^2^ against ALL reflections. The weighted *R*-factor *wR* and goodness of fit *S* are based on *F*^2^, conventional *R*-factors *R* are based on *F*, with *F* set to zero for negative *F*^2^. The threshold expression of *F*^2^ > 2σ(*F*^2^) is used only for calculating *R*-factors(gt) *etc*. and is not relevant to the choice of reflections for refinement. *R*-factors based on *F*^2^ are statistically about twice as large as those based on *F*, and *R*- factors based on ALL data will be even larger.

### Fractional atomic coordinates and isotropic or equivalent isotropic displacement parameters (Å^2^)

**Table:**

	*x*	*y*	*z*	*U*_iso_*/*U*_eq_	Occ. (<1)
Cd	0.70820 (3)	0.16101 (2)	0.698014 (15)	0.03567 (11)	
O1	0.3239 (4)	0.3694 (3)	1.4609 (2)	0.0702 (9)	
H1O	0.3520	0.3209	1.5122	0.105*	
O2	0.9918 (4)	0.3606 (3)	−0.19919 (18)	0.0599 (8)	
H2O	0.9745	0.3150	−0.2161	0.090*	
O3	0.9693 (3)	0.0674 (3)	0.72834 (19)	0.0504 (7)	
O4	0.9168 (3)	0.2304 (3)	0.73968 (19)	0.0488 (7)	
O5	1.4906 (4)	0.0739 (4)	0.7290 (2)	0.0762 (11)	
O6	1.4676 (3)	0.2372 (3)	0.6096 (2)	0.0619 (8)	
N1	0.6857 (3)	0.0720 (3)	0.85992 (19)	0.0357 (7)	
N2	0.5641 (3)	0.2916 (3)	0.7615 (2)	0.0412 (7)	
N3	0.5178 (4)	0.1543 (3)	1.1456 (2)	0.0439 (8)	
H3n	0.5437	0.0930	1.1924	0.053*	
N4	0.4233 (4)	0.3334 (3)	1.0646 (2)	0.0534 (9)	
N5	0.7763 (3)	0.0688 (3)	0.59460 (18)	0.0334 (6)	
N6	0.7974 (3)	0.2817 (3)	0.55281 (19)	0.0351 (7)	
N7	0.9021 (3)	0.1514 (3)	0.25644 (19)	0.0374 (7)	
H7n	0.9045	0.0920	0.2485	0.045*	
N8	0.9143 (4)	0.3264 (3)	0.2216 (2)	0.0436 (8)	
C1	0.7408 (4)	−0.0355 (4)	0.9060 (3)	0.0430 (9)	
H1	0.7878	−0.0793	0.8715	0.052*	
C2	0.7323 (4)	−0.0867 (4)	1.0031 (3)	0.0479 (10)	
H2	0.7702	−0.1630	1.0330	0.057*	
C3	0.6669 (4)	−0.0216 (4)	1.0534 (3)	0.0449 (9)	
H3	0.6608	−0.0535	1.1184	0.054*	
C4	0.6091 (4)	0.0922 (3)	1.0077 (2)	0.0370 (8)	
C5	0.6194 (4)	0.1374 (3)	0.9086 (2)	0.0352 (8)	
C6	0.5558 (4)	0.2550 (3)	0.8563 (2)	0.0377 (8)	
C7	0.5027 (5)	0.3966 (4)	0.7136 (3)	0.0533 (11)	
H7	0.5064	0.4209	0.6490	0.064*	
C8	0.4318 (5)	0.4735 (4)	0.7559 (3)	0.0594 (12)	
H8	0.3912	0.5475	0.7199	0.071*	
C9	0.4238 (5)	0.4376 (4)	0.8498 (3)	0.0589 (11)	
H9	0.3777	0.4871	0.8791	0.071*	
C10	0.4844 (4)	0.3266 (3)	0.9028 (3)	0.0452 (9)	
C11	0.4802 (4)	0.2799 (4)	1.0036 (3)	0.0446 (9)	
C12	0.5385 (4)	0.1685 (3)	1.0519 (2)	0.0406 (9)	
C13	0.4509 (5)	0.2526 (4)	1.1486 (2)	0.0449 (9)	
C14	0.4123 (5)	0.2773 (4)	1.2344 (3)	0.0459 (9)	
C15	0.4932 (4)	0.2150 (4)	1.3098 (3)	0.0470 (9)	
H15	0.5677	0.1533	1.3088	0.056*	
C16	0.4644 (4)	0.2436 (4)	1.3870 (3)	0.0472 (10)	
H16	0.5187	0.2008	1.4376	0.057*	
C17	0.3548 (5)	0.3359 (4)	1.3886 (3)	0.0470 (10)	
C18	0.2717 (5)	0.3975 (4)	1.3140 (3)	0.0552 (11)	
H18	0.1958	0.4582	1.3157	0.066*	
C19	0.3014 (5)	0.3691 (4)	1.2375 (3)	0.0553 (11)	
H19	0.2465	0.4119	1.1871	0.066*	
C20	0.7710 (4)	−0.0362 (3)	0.6174 (2)	0.0416 (9)	
H20	0.7461	−0.0792	0.6799	0.050*	
C21	0.8009 (5)	−0.0851 (3)	0.5527 (3)	0.0452 (9)	
H21	0.7968	−0.1593	0.5716	0.054*	
C22	0.8361 (4)	−0.0228 (3)	0.4611 (3)	0.0401 (8)	
H22	0.8560	−0.0541	0.4166	0.048*	
C23	0.8426 (4)	0.0893 (3)	0.4337 (2)	0.0333 (8)	
C24	0.8130 (3)	0.1325 (3)	0.5043 (2)	0.0297 (7)	
C25	0.8232 (4)	0.2460 (3)	0.4814 (2)	0.0299 (7)	
C26	0.8130 (5)	0.3828 (3)	0.5334 (3)	0.0455 (9)	
H26	0.7959	0.4068	0.5823	0.055*	
C27	0.8534 (5)	0.4553 (4)	0.4441 (3)	0.0530 (11)	
H27	0.8649	0.5254	0.4341	0.064*	
C28	0.8762 (5)	0.4227 (3)	0.3711 (3)	0.0466 (10)	
H28	0.9010	0.4709	0.3104	0.056*	
C29	0.8617 (4)	0.3158 (3)	0.3888 (2)	0.0357 (8)	
C30	0.8838 (4)	0.2720 (3)	0.3175 (2)	0.0363 (8)	
C31	0.8756 (4)	0.1642 (3)	0.3405 (2)	0.0327 (7)	
C32	0.9234 (4)	0.2507 (3)	0.1887 (2)	0.0392 (8)	
C33	0.9472 (4)	0.2732 (3)	0.0876 (2)	0.0404 (8)	
C34	1.0177 (5)	0.3571 (3)	0.0255 (3)	0.0502 (10)	
H34	1.0558	0.3953	0.0484	0.060*	
C35	1.0324 (5)	0.3849 (3)	−0.0700 (3)	0.0504 (10)	
H35	1.0810	0.4408	−0.1108	0.060*	
C36	0.9748 (5)	0.3292 (3)	−0.1050 (2)	0.0426 (9)	
C37	0.9070 (5)	0.2434 (4)	−0.0443 (3)	0.0491 (10)	
H37	0.8707	0.2045	−0.0675	0.059*	
C38	0.8935 (5)	0.2154 (4)	0.0518 (3)	0.0488 (10)	
H38	0.8481	0.1575	0.0926	0.059*	
C39	1.0066 (4)	0.1455 (4)	0.7328 (2)	0.0430 (9)	0.677 (15)
C40	1.1717 (6)	0.1176 (6)	0.7339 (4)	0.0345 (18)	0.677 (15)
H40	1.2180	0.0488	0.7754	0.041*	0.677 (15)
C41	1.2483 (6)	0.1893 (7)	0.6774 (4)	0.046 (2)	0.677 (15)
H41	1.2001	0.2602	0.6400	0.055*	0.677 (15)
C42	1.4171 (4)	0.1601 (5)	0.6706 (3)	0.0618 (14)	0.677 (15)
C39'	1.0066 (4)	0.1455 (4)	0.7328 (2)	0.0430 (9)	0.323 (15)
C40'	1.1567 (17)	0.1868 (16)	0.7021 (10)	0.039 (4)*	0.323 (15)
H40'	1.1611	0.2589	0.6913	0.047*	0.323 (15)
C41'	1.2730 (13)	0.1107 (13)	0.6934 (8)	0.032 (4)*	0.323 (15)
H41'	1.2727	0.0395	0.6994	0.038*	0.323 (15)
C42'	1.4171 (4)	0.1601 (5)	0.6706 (3)	0.0618 (14)	0.323 (15)

### Atomic displacement parameters (Å^2^)

**Table:**

	*U*^11^	*U*^22^	*U*^33^	*U*^12^	*U*^13^	*U*^23^
Cd	0.03240 (15)	0.0554 (2)	0.02243 (14)	−0.01454 (12)	0.00236 (9)	−0.01769 (12)
O1	0.094 (3)	0.069 (2)	0.0517 (18)	0.0083 (19)	−0.0146 (17)	−0.0412 (17)
O2	0.104 (3)	0.0573 (19)	0.0251 (13)	−0.0357 (18)	−0.0051 (14)	−0.0116 (13)
O3	0.0423 (15)	0.0675 (19)	0.0559 (17)	−0.0073 (14)	−0.0044 (12)	−0.0412 (15)
O4	0.0499 (16)	0.0595 (18)	0.0457 (15)	−0.0180 (15)	−0.0034 (12)	−0.0257 (14)
O5	0.0559 (19)	0.134 (3)	0.0345 (15)	−0.045 (2)	0.0013 (14)	−0.0170 (18)
O6	0.0412 (16)	0.097 (3)	0.0581 (19)	−0.0181 (17)	−0.0007 (14)	−0.0404 (19)
N1	0.0287 (14)	0.051 (2)	0.0278 (14)	−0.0112 (14)	0.0026 (11)	−0.0169 (14)
N2	0.0344 (16)	0.055 (2)	0.0274 (14)	−0.0065 (15)	−0.0044 (12)	−0.0113 (14)
N3	0.0433 (18)	0.056 (2)	0.0266 (15)	−0.0088 (16)	−0.0011 (13)	−0.0139 (15)
N4	0.062 (2)	0.059 (2)	0.0395 (18)	−0.0094 (18)	−0.0001 (16)	−0.0244 (17)
N5	0.0328 (15)	0.0449 (18)	0.0254 (13)	−0.0170 (14)	0.0011 (11)	−0.0129 (13)
N6	0.0369 (16)	0.0431 (18)	0.0307 (14)	−0.0094 (14)	0.0013 (12)	−0.0214 (13)
N7	0.0479 (18)	0.0415 (18)	0.0276 (14)	−0.0150 (15)	0.0007 (12)	−0.0170 (13)
N8	0.063 (2)	0.0420 (18)	0.0276 (15)	−0.0128 (16)	−0.0039 (14)	−0.0150 (14)
C1	0.038 (2)	0.053 (3)	0.0369 (19)	−0.0070 (18)	0.0016 (16)	−0.0211 (18)
C2	0.042 (2)	0.048 (2)	0.043 (2)	−0.0046 (18)	−0.0047 (17)	−0.0121 (19)
C3	0.040 (2)	0.060 (3)	0.0297 (18)	−0.0102 (19)	−0.0070 (15)	−0.0112 (18)
C4	0.0298 (17)	0.054 (2)	0.0289 (17)	−0.0135 (17)	0.0011 (14)	−0.0170 (17)
C5	0.0271 (16)	0.052 (2)	0.0261 (16)	−0.0117 (16)	0.0003 (13)	−0.0147 (16)
C6	0.0301 (17)	0.056 (2)	0.0286 (17)	−0.0101 (17)	0.0010 (14)	−0.0192 (17)
C7	0.046 (2)	0.065 (3)	0.034 (2)	−0.006 (2)	−0.0034 (17)	−0.011 (2)
C8	0.058 (3)	0.052 (3)	0.048 (2)	−0.001 (2)	−0.010 (2)	−0.006 (2)
C9	0.060 (3)	0.059 (3)	0.052 (2)	−0.005 (2)	0.000 (2)	−0.025 (2)
C10	0.042 (2)	0.052 (3)	0.0361 (19)	−0.0093 (19)	−0.0005 (16)	−0.0153 (18)
C11	0.045 (2)	0.058 (3)	0.0343 (19)	−0.0095 (19)	0.0024 (16)	−0.0259 (19)
C12	0.0386 (19)	0.055 (3)	0.0274 (17)	−0.0103 (18)	−0.0016 (14)	−0.0160 (17)
C13	0.051 (2)	0.063 (3)	0.0290 (18)	−0.018 (2)	0.0058 (16)	−0.0267 (18)
C14	0.050 (2)	0.058 (3)	0.0347 (19)	−0.015 (2)	0.0026 (16)	−0.0234 (18)
C15	0.043 (2)	0.057 (3)	0.045 (2)	−0.0060 (19)	0.0002 (17)	−0.029 (2)
C16	0.042 (2)	0.066 (3)	0.038 (2)	−0.010 (2)	−0.0048 (16)	−0.026 (2)
C17	0.052 (2)	0.058 (3)	0.041 (2)	−0.013 (2)	−0.0018 (17)	−0.0294 (19)
C18	0.061 (3)	0.057 (3)	0.050 (2)	0.001 (2)	−0.006 (2)	−0.032 (2)
C19	0.060 (3)	0.061 (3)	0.040 (2)	−0.002 (2)	−0.0129 (19)	−0.018 (2)
C20	0.047 (2)	0.049 (2)	0.0297 (17)	−0.0233 (19)	0.0057 (15)	−0.0120 (17)
C21	0.052 (2)	0.041 (2)	0.048 (2)	−0.0234 (19)	0.0017 (18)	−0.0165 (18)
C22	0.044 (2)	0.045 (2)	0.0398 (19)	−0.0158 (18)	0.0015 (16)	−0.0243 (17)
C23	0.0296 (17)	0.043 (2)	0.0311 (17)	−0.0100 (16)	−0.0006 (13)	−0.0185 (16)
C24	0.0243 (15)	0.041 (2)	0.0270 (15)	−0.0100 (15)	−0.0012 (12)	−0.0150 (15)
C25	0.0292 (16)	0.0350 (19)	0.0271 (16)	−0.0054 (15)	−0.0002 (13)	−0.0161 (14)
C26	0.060 (3)	0.047 (2)	0.0369 (19)	−0.016 (2)	0.0046 (17)	−0.0255 (18)
C27	0.081 (3)	0.039 (2)	0.043 (2)	−0.022 (2)	0.006 (2)	−0.0197 (18)
C28	0.066 (3)	0.039 (2)	0.0327 (19)	−0.016 (2)	0.0041 (17)	−0.0131 (17)
C29	0.041 (2)	0.038 (2)	0.0288 (17)	−0.0103 (16)	0.0004 (14)	−0.0150 (15)
C30	0.0419 (19)	0.041 (2)	0.0252 (16)	−0.0100 (17)	0.0003 (14)	−0.0143 (15)
C31	0.0366 (18)	0.039 (2)	0.0256 (16)	−0.0080 (16)	−0.0022 (13)	−0.0168 (15)
C32	0.045 (2)	0.046 (2)	0.0265 (17)	−0.0101 (18)	−0.0001 (14)	−0.0161 (16)
C33	0.048 (2)	0.045 (2)	0.0270 (17)	−0.0095 (18)	−0.0016 (15)	−0.0146 (16)
C34	0.079 (3)	0.047 (2)	0.0354 (19)	−0.026 (2)	−0.0023 (19)	−0.0202 (18)
C35	0.080 (3)	0.044 (2)	0.0317 (19)	−0.027 (2)	0.0051 (19)	−0.0153 (17)
C36	0.059 (2)	0.042 (2)	0.0254 (17)	−0.0114 (19)	0.0003 (16)	−0.0141 (16)
C37	0.057 (2)	0.066 (3)	0.0352 (19)	−0.023 (2)	−0.0017 (17)	−0.024 (2)
C38	0.058 (2)	0.061 (3)	0.0301 (18)	−0.031 (2)	0.0017 (17)	−0.0122 (18)
C39	0.0338 (19)	0.074 (3)	0.0337 (19)	−0.016 (2)	0.0024 (15)	−0.0331 (19)
C40	0.034 (3)	0.040 (4)	0.029 (3)	−0.009 (3)	−0.008 (2)	−0.011 (3)
C41	0.026 (3)	0.062 (6)	0.045 (3)	−0.017 (3)	−0.001 (2)	−0.014 (3)
C42	0.032 (2)	0.132 (5)	0.037 (2)	−0.028 (3)	0.0003 (17)	−0.043 (3)
C39'	0.0338 (19)	0.074 (3)	0.0337 (19)	−0.016 (2)	0.0024 (15)	−0.0331 (19)
C42'	0.032 (2)	0.132 (5)	0.037 (2)	−0.028 (3)	0.0003 (17)	−0.043 (3)

### Geometric parameters (Å, °)

**Table:**

Cd—N1	2.320 (3)	C9—H9	0.9300
Cd—N6	2.335 (3)	C10—C11	1.446 (5)
Cd—N5	2.343 (3)	C11—C12	1.374 (6)
Cd—N2	2.351 (3)	C13—C14	1.483 (5)
Cd—O3	2.496 (3)	C14—C15	1.384 (6)
Cd—O5^i^	2.541 (3)	C14—C19	1.390 (6)
Cd—O6^i^	2.671 (3)	C15—C16	1.388 (5)
Cd—O4	2.742 (3)	C15—H15	0.9300
O1—C17	1.353 (5)	C16—C17	1.382 (6)
O1—H1O	0.8400	C16—H16	0.9300
O2—C36	1.355 (4)	C17—C18	1.385 (6)
O2—H2O	0.8401	C18—C19	1.376 (6)
O3—C39'	1.246 (5)	C18—H18	0.9300
O3—C39	1.246 (5)	C19—H19	0.9300
O4—C39'	1.249 (5)	C20—C21	1.385 (5)
O4—C39	1.249 (5)	C20—H20	0.9300
O5—C42'	1.267 (6)	C21—C22	1.358 (5)
O5—C42	1.267 (6)	C21—H21	0.9300
O5—Cd^ii^	2.541 (3)	C22—C23	1.408 (5)
O6—C42'	1.220 (6)	C22—H22	0.9300
O6—C42	1.220 (6)	C23—C24	1.416 (5)
N1—C1	1.323 (5)	C23—C31	1.422 (5)
N1—C5	1.350 (5)	C24—C25	1.452 (5)
N2—C7	1.317 (5)	C25—C29	1.404 (5)
N2—C6	1.361 (4)	C26—C27	1.385 (6)
N3—C13	1.315 (5)	C26—H26	0.9300
N3—C12	1.393 (4)	C27—C28	1.364 (5)
N3—H3n	0.8600	C27—H27	0.9300
N4—C13	1.339 (5)	C28—C29	1.398 (5)
N4—C11	1.376 (5)	C28—H28	0.9300
N5—C20	1.325 (5)	C29—C30	1.439 (5)
N5—C24	1.351 (4)	C30—C31	1.370 (5)
N6—C26	1.318 (5)	C32—C33	1.479 (5)
N6—C25	1.363 (4)	C33—C34	1.385 (6)
N7—C32	1.357 (5)	C33—C38	1.396 (5)
N7—C31	1.379 (4)	C34—C35	1.385 (5)
N7—H7n	0.8600	C34—H34	0.9300
N8—C32	1.312 (5)	C35—C36	1.385 (5)
N8—C30	1.386 (4)	C35—H35	0.9300
C1—C2	1.391 (5)	C36—C37	1.383 (6)
C1—H1	0.9300	C37—C38	1.393 (5)
C2—C3	1.365 (6)	C37—H37	0.9300
C2—H2	0.9300	C38—H38	0.9300
C3—C4	1.392 (6)	C39—C40	1.513 (6)
C3—H3	0.9300	C40—C41	1.292 (12)
C4—C5	1.418 (5)	C40—H40	0.9300
C4—C12	1.421 (5)	C41—C42	1.535 (7)
C5—C6	1.460 (5)	C41—H41	0.9300
C6—C10	1.398 (5)	C39'—C40'	1.593 (15)
C7—C8	1.406 (7)	C40'—C41'	1.30 (3)
C7—H7	0.9300	C40'—H40'	0.9300
C8—C9	1.350 (6)	C41'—C42'	1.611 (12)
C8—H8	0.9300	C41'—H41'	0.9300
C9—C10	1.390 (6)		
			
N1—Cd—N6	155.24 (10)	C15—C14—C19	118.7 (4)
N1—Cd—N5	124.62 (11)	C15—C14—C13	120.8 (4)
N6—Cd—N5	71.47 (10)	C19—C14—C13	120.4 (4)
N1—Cd—N2	71.63 (11)	C14—C15—C16	120.7 (4)
N6—Cd—N2	100.10 (10)	C14—C15—H15	119.6
N5—Cd—N2	156.65 (10)	C16—C15—H15	119.6
N1—Cd—O3	78.07 (10)	C17—C16—C15	119.9 (4)
N6—Cd—O3	87.88 (10)	C17—C16—H16	120.0
N5—Cd—O3	79.62 (9)	C15—C16—H16	120.0
N2—Cd—O3	122.59 (9)	O1—C17—C16	122.6 (4)
N1—Cd—O5^i^	78.38 (10)	O1—C17—C18	117.7 (4)
N6—Cd—O5^i^	125.81 (10)	C16—C17—C18	119.7 (4)
N5—Cd—O5^i^	77.89 (11)	C19—C18—C17	120.1 (4)
N2—Cd—O5^i^	90.86 (12)	C19—C18—H18	119.9
O3—Cd—O5^i^	129.15 (12)	C17—C18—H18	119.9
N1—Cd—O6^i^	119.07 (10)	C18—C19—C14	120.9 (4)
N6—Cd—O6^i^	80.39 (10)	C18—C19—H19	119.6
N5—Cd—O6^i^	78.73 (10)	C14—C19—H19	119.6
N2—Cd—O6^i^	78.37 (10)	N5—C20—C21	123.2 (3)
O3—Cd—O6^i^	157.74 (9)	N5—C20—H20	118.4
O5^i^—Cd—O6^i^	50.03 (11)	C21—C20—H20	118.4
N1—Cd—O4	79.34 (9)	C22—C21—C20	118.9 (3)
N6—Cd—O4	76.05 (9)	C22—C21—H21	120.5
N5—Cd—O4	119.50 (9)	C20—C21—H21	120.5
N2—Cd—O4	77.52 (10)	C21—C22—C23	119.8 (3)
O3—Cd—O4	49.28 (9)	C21—C22—H22	120.1
O5^i^—Cd—O4	157.20 (9)	C23—C22—H22	120.1
O6^i^—Cd—O4	142.46 (10)	C22—C23—C24	117.8 (3)
C17—O1—H1O	116.3	C22—C23—C31	125.7 (3)
C36—O2—H2O	113.5	C24—C23—C31	116.5 (3)
C39'—O3—C39	0.0 (3)	N5—C24—C23	120.9 (3)
C39'—O3—Cd	98.9 (2)	N5—C24—C25	118.4 (3)
C39—O3—Cd	98.9 (2)	C23—C24—C25	120.6 (3)
C39'—O4—C39	0.0 (3)	N6—C25—C29	121.2 (3)
C39'—O4—Cd	87.2 (2)	N6—C25—C24	117.9 (3)
C39—O4—Cd	87.2 (2)	C29—C25—C24	120.9 (3)
C42'—O5—C42	0.0 (5)	N6—C26—C27	123.6 (3)
C42'—O5—Cd^ii^	93.9 (3)	N6—C26—H26	118.2
C42—O5—Cd^ii^	93.9 (3)	C27—C26—H26	118.2
C42'—O6—C42	0.0 (8)	C28—C27—C26	119.2 (4)
C1—N1—C5	119.1 (3)	C28—C27—H27	120.4
C1—N1—Cd	124.2 (2)	C26—C27—H27	120.4
C5—N1—Cd	116.6 (2)	C27—C28—C29	119.0 (3)
C7—N2—C6	118.8 (3)	C27—C28—H28	120.5
C7—N2—Cd	126.0 (3)	C29—C28—H28	120.5
C6—N2—Cd	115.0 (2)	C28—C29—C25	118.6 (3)
C13—N3—C12	106.8 (3)	C28—C29—C30	124.0 (3)
C13—N3—H3n	126.6	C25—C29—C30	117.4 (3)
C12—N3—H3n	126.6	C31—C30—N8	111.1 (3)
C13—N4—C11	103.9 (3)	C31—C30—C29	120.8 (3)
C20—N5—C24	119.3 (3)	N8—C30—C29	128.1 (3)
C20—N5—Cd	124.9 (2)	C30—C31—N7	105.1 (3)
C24—N5—Cd	115.6 (2)	C30—C31—C23	123.6 (3)
C26—N6—C25	118.4 (3)	N7—C31—C23	131.3 (3)
C26—N6—Cd	125.7 (2)	N8—C32—N7	113.3 (3)
C25—N6—Cd	115.6 (2)	N8—C32—C33	123.2 (3)
C32—N7—C31	106.6 (3)	N7—C32—C33	123.4 (3)
C32—N7—H7n	126.7	C34—C33—C38	118.5 (3)
C31—N7—H7n	126.7	C34—C33—C32	120.0 (3)
C32—N8—C30	103.9 (3)	C38—C33—C32	121.5 (3)
N1—C1—C2	123.6 (4)	C35—C34—C33	121.1 (4)
N1—C1—H1	118.2	C35—C34—H34	119.5
C2—C1—H1	118.2	C33—C34—H34	119.5
C3—C2—C1	118.1 (4)	C34—C35—C36	120.0 (4)
C3—C2—H2	121.0	C34—C35—H35	120.0
C1—C2—H2	121.0	C36—C35—H35	120.0
C2—C3—C4	120.2 (3)	O2—C36—C37	122.7 (3)
C2—C3—H3	119.9	O2—C36—C35	117.3 (3)
C4—C3—H3	119.9	C37—C36—C35	120.0 (3)
C3—C4—C5	118.2 (3)	C36—C37—C38	119.7 (4)
C3—C4—C12	125.6 (3)	C36—C37—H37	120.2
C5—C4—C12	116.2 (3)	C38—C37—H37	120.2
N1—C5—C4	120.8 (3)	C37—C38—C33	120.7 (4)
N1—C5—C6	118.0 (3)	C37—C38—H38	119.6
C4—C5—C6	121.1 (3)	C33—C38—H38	119.6
N2—C6—C10	121.3 (3)	O3—C39—O4	123.3 (3)
N2—C6—C5	118.0 (3)	O3—C39—C40	109.8 (4)
C10—C6—C5	120.7 (3)	O4—C39—C40	126.9 (4)
N2—C7—C8	122.8 (4)	C41—C40—C39	120.1 (7)
N2—C7—H7	118.6	C41—C40—H40	119.9
C8—C7—H7	118.6	C39—C40—H40	119.9
C9—C8—C7	118.5 (4)	C40—C41—C42	121.5 (7)
C9—C8—H8	120.7	C40—C41—H41	119.2
C7—C8—H8	120.7	C42—C41—H41	119.2
C8—C9—C10	120.2 (4)	O6—C42—O5	125.1 (4)
C8—C9—H9	119.9	O6—C42—C41	110.8 (5)
C10—C9—H9	119.9	O5—C42—C41	123.5 (5)
C9—C10—C6	118.4 (4)	O3—C39'—O4	123.3 (3)
C9—C10—C11	124.4 (4)	O3—C39'—C40'	133.1 (7)
C6—C10—C11	117.2 (4)	O4—C39'—C40'	101.1 (7)
C12—C11—N4	110.3 (3)	C41'—C40'—C39'	114.1 (14)
C12—C11—C10	121.3 (3)	C41'—C40'—H40'	123.0
N4—C11—C10	128.4 (4)	C39'—C40'—H40'	123.0
C11—C12—N3	105.4 (3)	C40'—C41'—C42'	108.8 (14)
C11—C12—C4	123.4 (3)	C40'—C41'—H41'	125.6
N3—C12—C4	131.1 (4)	C42'—C41'—H41'	125.6
N3—C13—N4	113.6 (3)	O6—C42'—O5	125.1 (4)
N3—C13—C14	125.3 (4)	O6—C42'—C41'	138.0 (6)
N4—C13—C14	121.1 (4)	O5—C42'—C41'	95.3 (6)
			
N1—Cd—O3—C39'	−92.7 (2)	C13—N3—C12—C4	177.4 (4)
N6—Cd—O3—C39'	66.8 (2)	C3—C4—C12—C11	−178.4 (4)
N5—Cd—O3—C39'	138.3 (2)	C5—C4—C12—C11	1.0 (5)
N2—Cd—O3—C39'	−33.9 (3)	C3—C4—C12—N3	4.9 (6)
O5^i^—Cd—O3—C39'	−156.8 (2)	C5—C4—C12—N3	−175.7 (4)
O6^i^—Cd—O3—C39'	124.7 (3)	C12—N3—C13—N4	0.3 (5)
O4—Cd—O3—C39'	−6.6 (2)	C12—N3—C13—C14	−177.5 (4)
N1—Cd—O3—C39	−92.7 (2)	C11—N4—C13—N3	−0.7 (5)
N6—Cd—O3—C39	66.8 (2)	C11—N4—C13—C14	177.2 (4)
N5—Cd—O3—C39	138.3 (2)	N3—C13—C14—C15	27.2 (6)
N2—Cd—O3—C39	−33.9 (3)	N4—C13—C14—C15	−150.5 (4)
O5^i^—Cd—O3—C39	−156.8 (2)	N3—C13—C14—C19	−158.0 (4)
O6^i^—Cd—O3—C39	124.7 (3)	N4—C13—C14—C19	24.4 (6)
O4—Cd—O3—C39	−6.6 (2)	C19—C14—C15—C16	−0.1 (6)
N1—Cd—O4—C39'	89.9 (2)	C13—C14—C15—C16	174.9 (4)
N6—Cd—O4—C39'	−92.9 (2)	C14—C15—C16—C17	−0.6 (6)
N5—Cd—O4—C39'	−34.0 (2)	C15—C16—C17—O1	−178.4 (4)
N2—Cd—O4—C39'	163.2 (2)	C15—C16—C17—C18	1.7 (6)
O3—Cd—O4—C39'	6.5 (2)	O1—C17—C18—C19	178.1 (4)
O5^i^—Cd—O4—C39'	102.3 (4)	C16—C17—C18—C19	−2.1 (7)
O6^i^—Cd—O4—C39'	−145.6 (2)	C17—C18—C19—C14	1.4 (7)
N1—Cd—O4—C39	89.9 (2)	C15—C14—C19—C18	−0.3 (7)
N6—Cd—O4—C39	−92.9 (2)	C13—C14—C19—C18	−175.3 (4)
N5—Cd—O4—C39	−34.0 (2)	C24—N5—C20—C21	−0.3 (5)
N2—Cd—O4—C39	163.2 (2)	Cd—N5—C20—C21	174.6 (3)
O3—Cd—O4—C39	6.5 (2)	N5—C20—C21—C22	−0.6 (6)
O5^i^—Cd—O4—C39	102.3 (4)	C20—C21—C22—C23	0.3 (6)
O6^i^—Cd—O4—C39	−145.6 (2)	C21—C22—C23—C24	0.7 (5)
N6—Cd—N1—C1	−108.9 (3)	C21—C22—C23—C31	−178.9 (4)
N5—Cd—N1—C1	16.1 (3)	C20—N5—C24—C23	1.3 (5)
N2—Cd—N1—C1	177.3 (3)	Cd—N5—C24—C23	−174.0 (2)
O3—Cd—N1—C1	−52.2 (3)	C20—N5—C24—C25	−177.9 (3)
O5^i^—Cd—N1—C1	82.4 (3)	Cd—N5—C24—C25	6.8 (4)
O6^i^—Cd—N1—C1	112.6 (3)	C22—C23—C24—N5	−1.5 (5)
O4—Cd—N1—C1	−102.5 (3)	C31—C23—C24—N5	178.1 (3)
N6—Cd—N1—C5	67.0 (4)	C22—C23—C24—C25	177.7 (3)
N5—Cd—N1—C5	−168.0 (2)	C31—C23—C24—C25	−2.7 (5)
N2—Cd—N1—C5	−6.8 (2)	C26—N6—C25—C29	−1.6 (5)
O3—Cd—N1—C5	123.7 (2)	Cd—N6—C25—C29	173.1 (3)
O5^i^—Cd—N1—C5	−101.7 (2)	C26—N6—C25—C24	176.9 (3)
O6^i^—Cd—N1—C5	−71.5 (3)	Cd—N6—C25—C24	−8.4 (4)
O4—Cd—N1—C5	73.4 (2)	N5—C24—C25—N6	1.0 (4)
N1—Cd—N2—C7	−178.2 (3)	C23—C24—C25—N6	−178.2 (3)
N6—Cd—N2—C7	26.0 (3)	N5—C24—C25—C29	179.6 (3)
N5—Cd—N2—C7	−40.3 (5)	C23—C24—C25—C29	0.4 (5)
O3—Cd—N2—C7	119.9 (3)	C25—N6—C26—C27	0.3 (6)
O5^i^—Cd—N2—C7	−100.7 (3)	Cd—N6—C26—C27	−173.8 (3)
O6^i^—Cd—N2—C7	−51.9 (3)	N6—C26—C27—C28	1.3 (7)
O4—Cd—N2—C7	99.1 (3)	C26—C27—C28—C29	−1.6 (7)
N1—Cd—N2—C6	7.5 (2)	C27—C28—C29—C25	0.4 (6)
N6—Cd—N2—C6	−148.4 (2)	C27—C28—C29—C30	−179.6 (4)
N5—Cd—N2—C6	145.3 (3)	N6—C25—C29—C28	1.3 (5)
O3—Cd—N2—C6	−54.4 (3)	C24—C25—C29—C28	−177.3 (3)
O5^i^—Cd—N2—C6	84.9 (3)	N6—C25—C29—C30	−178.7 (3)
O6^i^—Cd—N2—C6	133.7 (3)	C24—C25—C29—C30	2.7 (5)
O4—Cd—N2—C6	−75.3 (2)	C32—N8—C30—C31	0.2 (4)
N1—Cd—N5—C20	18.2 (3)	C32—N8—C30—C29	−179.7 (4)
N6—Cd—N5—C20	177.0 (3)	C28—C29—C30—C31	176.4 (4)
N2—Cd—N5—C20	−111.1 (3)	C25—C29—C30—C31	−3.6 (5)
O3—Cd—N5—C20	85.7 (3)	C28—C29—C30—N8	−3.7 (6)
O5^i^—Cd—N5—C20	−48.3 (3)	C25—C29—C30—N8	176.3 (4)
O6^i^—Cd—N5—C20	−99.5 (3)	N8—C30—C31—N7	0.1 (4)
O4—Cd—N5—C20	115.8 (3)	C29—C30—C31—N7	180.0 (3)
N1—Cd—N5—C24	−166.8 (2)	N8—C30—C31—C23	−178.6 (3)
N6—Cd—N5—C24	−8.0 (2)	C29—C30—C31—C23	1.3 (6)
N2—Cd—N5—C24	64.0 (4)	C32—N7—C31—C30	−0.3 (4)
O3—Cd—N5—C24	−99.2 (2)	C32—N7—C31—C23	178.2 (4)
O5^i^—Cd—N5—C24	126.7 (2)	C22—C23—C31—C30	−178.5 (3)
O6^i^—Cd—N5—C24	75.5 (2)	C24—C23—C31—C30	1.9 (5)
O4—Cd—N5—C24	−69.2 (2)	C22—C23—C31—N7	3.2 (6)
N1—Cd—N6—C26	−42.5 (5)	C24—C23—C31—N7	−176.4 (3)
N5—Cd—N6—C26	−177.2 (3)	C30—N8—C32—N7	−0.4 (4)
N2—Cd—N6—C26	25.3 (3)	C30—N8—C32—C33	176.7 (3)
O3—Cd—N6—C26	−97.5 (3)	C31—N7—C32—N8	0.5 (4)
O5^i^—Cd—N6—C26	123.7 (3)	C31—N7—C32—C33	−176.6 (3)
O6^i^—Cd—N6—C26	101.5 (3)	N8—C32—C33—C34	25.9 (6)
O4—Cd—N6—C26	−49.0 (3)	N7—C32—C33—C34	−157.3 (4)
N1—Cd—N6—C25	143.2 (3)	N8—C32—C33—C38	−150.9 (4)
N5—Cd—N6—C25	8.5 (2)	N7—C32—C33—C38	25.9 (6)
N2—Cd—N6—C25	−149.0 (2)	C38—C33—C34—C35	1.3 (7)
O3—Cd—N6—C25	88.3 (2)	C32—C33—C34—C35	−175.6 (4)
O5^i^—Cd—N6—C25	−50.5 (3)	C33—C34—C35—C36	0.7 (7)
O6^i^—Cd—N6—C25	−72.7 (2)	C34—C35—C36—O2	179.6 (4)
O4—Cd—N6—C25	136.7 (3)	C34—C35—C36—C37	−2.2 (7)
C5—N1—C1—C2	1.1 (5)	O2—C36—C37—C38	179.9 (4)
Cd—N1—C1—C2	176.9 (3)	C35—C36—C37—C38	1.8 (7)
N1—C1—C2—C3	−1.7 (6)	C36—C37—C38—C33	0.2 (7)
C1—C2—C3—C4	0.8 (6)	C34—C33—C38—C37	−1.7 (7)
C2—C3—C4—C5	0.5 (5)	C32—C33—C38—C37	175.1 (4)
C2—C3—C4—C12	180.0 (4)	C39'—O3—C39—O4	0(46)
C1—N1—C5—C4	0.3 (5)	Cd—O3—C39—O4	13.2 (4)
Cd—N1—C5—C4	−175.8 (2)	C39'—O3—C39—C40	0(100)
C1—N1—C5—C6	−178.3 (3)	Cd—O3—C39—C40	−170.0 (3)
Cd—N1—C5—C6	5.6 (4)	C39'—O4—C39—O3	0(35)
C3—C4—C5—N1	−1.1 (5)	Cd—O4—C39—O3	−11.9 (4)
C12—C4—C5—N1	179.4 (3)	C39'—O4—C39—C40	0(100)
C3—C4—C5—C6	177.4 (3)	Cd—O4—C39—C40	171.8 (4)
C12—C4—C5—C6	−2.1 (5)	O3—C39—C40—C41	130.4 (6)
C7—N2—C6—C10	−0.3 (5)	O4—C39—C40—C41	−52.9 (7)
Cd—N2—C6—C10	174.5 (3)	C39—C40—C41—C42	−174.6 (4)
C7—N2—C6—C5	177.7 (3)	C42'—O6—C42—O5	0(100)
Cd—N2—C6—C5	−7.5 (4)	C42'—O6—C42—C41	0(27)
N1—C5—C6—N2	1.4 (5)	C42'—O5—C42—O6	0(100)
C4—C5—C6—N2	−177.2 (3)	Cd^ii^—O5—C42—O6	16.1 (5)
N1—C5—C6—C10	179.4 (3)	C42'—O5—C42—C41	0(19)
C4—C5—C6—C10	0.8 (5)	Cd^ii^—O5—C42—C41	−154.0 (5)
C6—N2—C7—C8	1.5 (6)	C40—C41—C42—O6	176.2 (6)
Cd—N2—C7—C8	−172.6 (3)	C40—C41—C42—O5	−12.4 (9)
N2—C7—C8—C9	−1.3 (7)	C39—O3—C39'—O4	0(46)
C7—C8—C9—C10	−0.2 (7)	Cd—O3—C39'—O4	13.2 (4)
C8—C9—C10—C6	1.4 (7)	C39—O3—C39'—C40'	0(100)
C8—C9—C10—C11	−179.3 (4)	Cd—O3—C39'—C40'	−145.2 (8)
N2—C6—C10—C9	−1.1 (6)	C39—O4—C39'—O3	0(35)
C5—C6—C10—C9	−179.0 (4)	Cd—O4—C39'—O3	−11.9 (4)
N2—C6—C10—C11	179.5 (3)	C39—O4—C39'—C40'	0(100)
C5—C6—C10—C11	1.6 (5)	Cd—O4—C39'—C40'	152.2 (5)
C13—N4—C11—C12	0.9 (5)	O3—C39'—C40'—C41'	−21.0 (16)
C13—N4—C11—C10	−178.6 (4)	O4—C39'—C40'—C41'	177.3 (10)
C9—C10—C11—C12	178.0 (4)	C39'—C40'—C41'—C42'	−175.4 (7)
C6—C10—C11—C12	−2.6 (6)	C42—O6—C42'—O5	0(100)
C9—C10—C11—N4	−2.6 (7)	C42—O6—C42'—C41'	0(20)
C6—C10—C11—N4	176.8 (4)	C42—O5—C42'—O6	0(100)
N4—C11—C12—N3	−0.7 (4)	Cd^ii^—O5—C42'—O6	16.1 (5)
C10—C11—C12—N3	178.8 (3)	C42—O5—C42'—C41'	0(24)
N4—C11—C12—C4	−178.1 (3)	Cd^ii^—O5—C42'—C41'	−176.4 (5)
C10—C11—C12—C4	1.4 (6)	C40'—C41'—C42'—O6	−61.2 (13)
C13—N3—C12—C11	0.3 (4)	C40'—C41'—C42'—O5	134.2 (10)

Symmetry codes: (i) *x*−1, *y*, *z*; (ii) *x*+1, *y*, *z*.

### Hydrogen-bond geometry (Å, °)

**Table:**

*D*—H···*A*	*D*—H	H···*A*	*D*···*A*	*D*—H···*A*
O1—H1O···O6^iii^	0.84	1.86	2.659 (5)	161
O2—H2O···O4^iv^	0.84	1.83	2.663 (5)	172
N3—H3n···O5^v^	0.86	2.15	2.893 (6)	144
N7—H7n···O3^vi^	0.86	2.06	2.785 (6)	141
C3—H3···O5^v^	0.93	2.48	3.309 (5)	148
C22—H22···O3^vi^	0.93	2.57	3.360 (5)	143
C28—H28···O2^vii^	0.93	2.55	3.390 (6)	150
C2—H2···Cg1^vi^	0.93	2.75	3.445 (5)	133
C21—H21···Cg2^viii^	0.93	2.79	3.554 (5)	140

Symmetry codes: (iii) *x*−1, *y*, *z*+1; (iv) *x*, *y*, *z*−1; (v) −*x*+2, −*y*, −*z*+2; (vi) −*x*+2, −*y*, −*z*+1; (vii) −*x*+2, −*y*+1, −*z*; (viii) −*x*+1, −*y*, −*z*+2.
